# Statistics of Amputations

**Published:** 1850-11

**Authors:** 


*Statistics of Amputations.* The Boston Medical Journal contains an invaluable paper, by Dr. George Hayward, exhibiting in detail the statistics of the amputations of large limbs, performed at the Massachusetts General Hospital from 1822 to 1849, inclusive—a period of twenty-seven years.

The following summary of the whole is thus stated:

"It appears, then, from these tables, that the whole number of amputations of large limbs that have ever been performed at the Hospital, is 146, on 141 patients; of this number, 32 died.

"Eighty-five had their limbs removed in consequence of disease, of whom 10 died.

"Fifty-six in consequence of injury; of whom 22 died; being one in 8½ of the former, and more than 1 in 3 of the latter.

| | | |
|---|---|---|
| 69 | patients had the thigh amputated | —19 died. |
| 50 | had the leg removed below the knee | —10 died. |
| 11 | had amputation above the elbow | — 1 died. |
| 11 | " below " | — 2 died. |
| 141 | | 32 |

"The ages of the patients were as follows:

| | | | | |
|---|---|---|---|---|
| Under | 20 years of age, | 26, | of whom | 4 died. |
| Between | 20 and 30 | 56, | " | 11 died. |
| " | 30 and 40 | 28, | " | 10 died. |
| " | 40 and 50 | 18, | " | 5 died. |
| " | 50 and 60 | 7, | " | 1 died. |
| " | 60 and 70 | 4, | " | 1 died. |
| | Over 70 | 2, | " | 0 died. |
| | | 741 | | 32 " |

We observe that Dr. Hayward renders his frank tribute to conservative surgery by the concession that "*amputation is often performed, when it might have been avoided,*" and adds: "It is difficult in many cases to decide on the best course; but *the operation should not be done without the clearest evidence of its necessity,* for it is a hazardous and painful one, and, even when perfectly successful, leaves the patient in a mutilated state." He expresses the fear, that amputation is often resorted to earlier than it should be, particularly in recent injuries; and thus accounts for its greater fatality, and rebukes hasty surgery. These sentiments do equal honor to his head and heart.—*N. Y. Med. Jour.*

